# Neuralgic Pill

**Published:** 1871-01

**Authors:** 


Neuralgic Pill.—Dr. T. C. Osborne says that the subjoined combination is very effectual in cases of neuralgia: zinci cyaruretum, gr. vj; quinæ sulphas, gr. ix; morphiæ sulphas, gr. iss; ext. belladonnæ, gr. iij; Ft. pilulæ, No. vj. S. One pill every six hours, until the pain is relieved.—*New Orleans Journal of Medicine.*

